# Length-dependent gene misexpression is associated with Alzheimer’s disease progression

**DOI:** 10.1038/s41598-017-00250-4

**Published:** 2017-03-15

**Authors:** Shahar Barbash, Thomas P. Sakmar

**Affiliations:** 1 Laboratory of Chemical Biology and Signal Transduction, The Rockefeller University, 1230 York Ave., New York, NY 10065 USA; 2 Department of Neurobiology, Care Sciences and Society, Division for Neurogeriatrics, Center for Alzheimer Research, Karolinska Institutet, 141 57 Huddinge, Sweden

## Abstract

Recent reports show transcription preference for long genes in neuronal tissues compared with non-neuronal tissues, and a gene-length dependent change in expression in the neurodevelopmental disease Rett syndrome (RTT). Whether the gene-length dependent changes in expression seen in RTT might also be seen in neurodegenerative diseases is not yet known. However, a reasonable hypothesis is that similar effects might be seen in neurodegenerative diseases as well as in RTT since a common general feature of both illnesses involves progressive dysfunction of synapses. Here, we demonstrate a clear length-dependent gene misexpression in the most prevalent neurodegenerative disease, Alzheimer’s disease. We show that the effect is associated with disease progression and can be attributed specifically to neurons. In particular, we observed gene length-dependent down regulation on the level of the whole tissue and gene length-dependent up regulation on the level of single cells. Our analysis shows that a gene-length effect on expression can be found in degenerative neurological illnesses, such as Alzheimer’s disease. Additional investigation to elucidate the precise mechanism underlying gene-length dependent changes in expression is warranted.

## Introduction

A recent study by Gabel *et al.* ^1^ showed misexpression of long genes in the Rett syndrome (RTT), a devastating neurodevelopmental disease caused mainly by mutations in the gene for *MECP2* (methyl CpG binding protein 2)^2^. Evidence was presented for the involvement of DNA-methylation in this process. To the best of our knowledge, this is the only study as of today that shows misexpression of long genes in a human brain condition. Given that the literature on length-dependent gene expression points at increased expression of long genes in neurons^3, 4^ and that neurons die in neurodegenerative diseases^5^, we hypothesized that this effect should also be evident in neurodegeneration in addition to neurodevelopmental conditions. Further support for this hypothesis also comes from findings on a class of enzymes that relieve supercoiling during DNA transcription, for example topoisomerases. These enzymes are associated with synaptic function^6, 7^ and are implicated in a variety of brain disorders including neurodegenerative disorders^8–10^. To investigate the involvement of this effect in neurodegeneration, we studied available datasets of the most common neurodegenerative disease, Alzheimer’s disease (AD).

In principle, length-dependent gene expression effects might be measured at the level of whole tissues, or at the level of specific cell populations within a tissue. The former usually, though not necessarily, points at a difference in cell composition while the latter points at different transcriptional characteristics of one cell type compared to another.

Still no clear statistical framework exists to measure gene-length dependent effects in samples. We therefore implemented two statistical approaches in analyzing our observations. We compared the 95% confidence intervals of Bootstrap resampling with replacement for the measured data and performed a threshold-dependent analysis that calls for differentially expressed genes. Our results show clear gene-length dependent misexpression in AD at both the whole tissue level and the cell specific levels and clear association with disease progression.

## Results and Discussion

**Figure 1:**
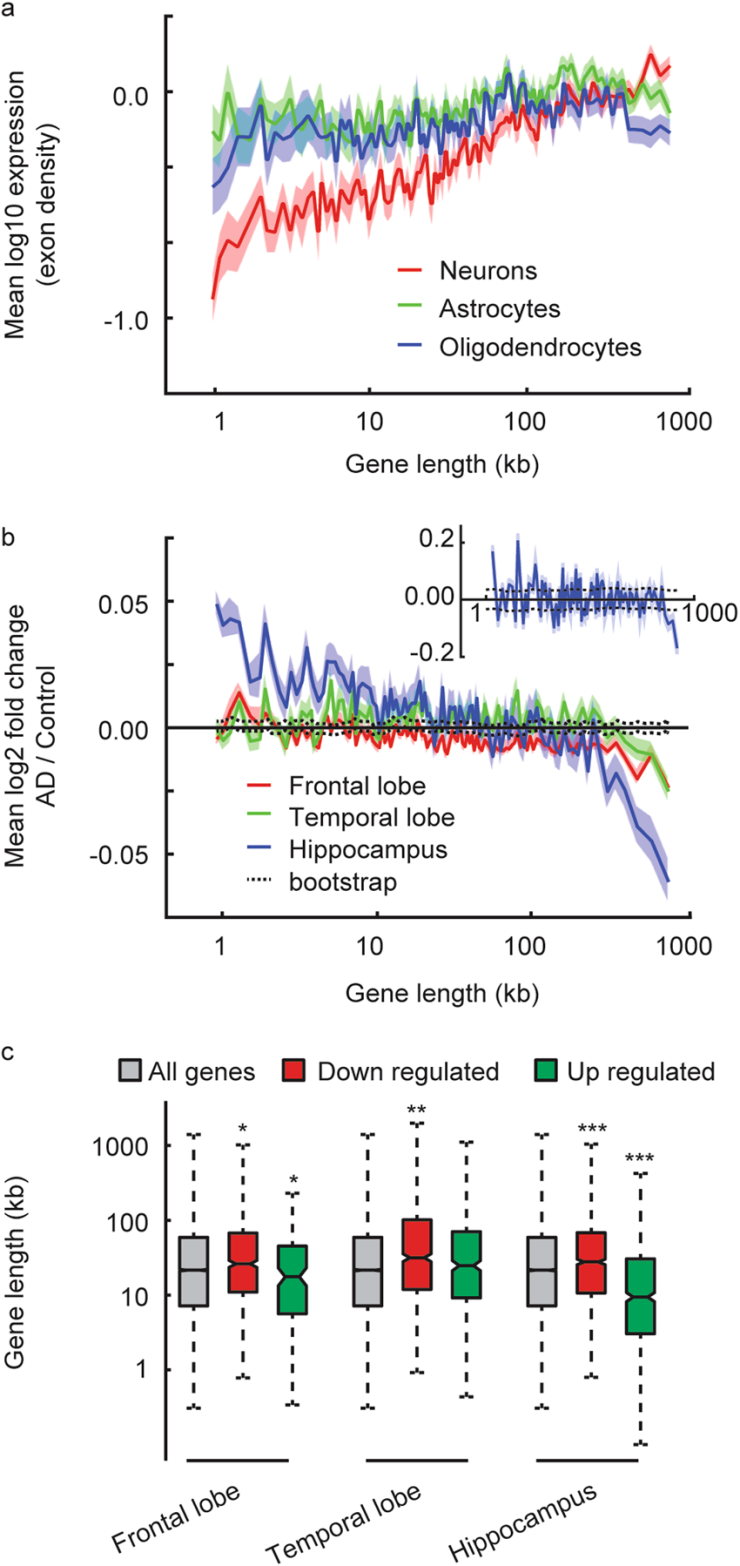
Length-dependent effect on expression associated with Alzheimer’s disease progression. (**a**) Mean expression of genes (log10 transformed) binned according to gene length for neurons, astrocytes and oligodendrocytes from human adult brain (n = 466 cells in total). Lines indicate mean expression for genes within each 200 gene bin (see Methods) and the ribbons represent the s.e.m. of each bin. (**b**) Mean expression changes of genes bind according to length as a function of gene length for human adult brain frontal lobe, temporal lobe and hippocampus (n = 79 samples in total). Lines and ribbons as in (**a**). Dashed black lines represent upper and lower 95% confidence intervals of 100 bootstrap iterations (see Methods). Inset shows similar effect for hippocampal samples of a different study (GSE1297, n = 16). (**c**) Boxplots showing the median (line), second to third quartiles (box), 1.5× the interquartile range (whiskers), and 1.58× the interquartile range/(√ number of genes) of gene lengths for gene detected as up- or down-regulated in AD frontal or temporal lobes or Hippocampus. Asterisks denote P < 0.05, P < 0.01 or P < 0.001 for two sample t-test of up-or down-regulated genes versus all genes, Bonferroni correction.

While previous studies reported a gene-length dependent expression based on analysis of whole tissues from mouse and human samples, and separately based on single-cell analysis from cell lines^1, 4^, we searched for this effect in specific cell populations from adult human brain dataset that includes 466 cells (GSE67835^11^), to examine the transcriptome of single cells in the context of the tissue. We identified a clear length-dependent increased expression specifically for neurons (Fig. 1a). This human-based cell-type specific approach serves to extend and deepen the mentioned whole tissue-based and the cell line-based studies.

We next examined the genome-wide change in gene expression as a function of gene length in AD samples in which cell death is most robust and widespread similar to Gabel *et al.* ^1^. We analyzed 79 AD samples from three different brain regions (GSE36980^12^). We identified length-dependent decreased expression in all three regions, with a more pronounced effect for hippocampus (Fig. 1b), which is generally the first region to be affected in AD. In order to attribute statistical significance to the observed effect we randomly shuffled across the gene lengths and their expression level and repeated the same analysis as before. This was performed 100 times to produce a bootstrap distribution (black dashed lines in Fig. 1b). Comparing the results to the bootstrap 95% confidence intervals suggests statistical significant effect for all examined brain regions with a much larger distance between the bootstrap data and those of the hippocampus than for the other two regions. Therefore, the length-dependent decreased expression was observed to be associated with neurodegeneration and tends to correlate with disease progression.

In order to further validate the observed association with an independent data set, we analyzed additional expression data files from human hippocampus (GSE1297^13^). Again, we observed a similar effect of gene length dependent down regulation, although in this case the association was most robust for the top four gene length groups. Taking a threshold-dependent approach and examining gene lengths for genes detected as either up- or down-regulated in AD brain regions (t-test P < 0.01, no multiple testing correction) showed a similar effect (Fig. 1c). Similar analysis was performed before in data sets from mouse tissue, and failed to detect length-dependent misexpression in AD^1^. This negative result is probably because it was based on an animal model for AD in which neurodegeneration is sparse to nonexistent (GSE56772), as is the case in most of AD mouse models^14^. The difficulty of making conclusions about a human disease based on animal models has been raised before in the context of AD^14^.

**Figure 2:**
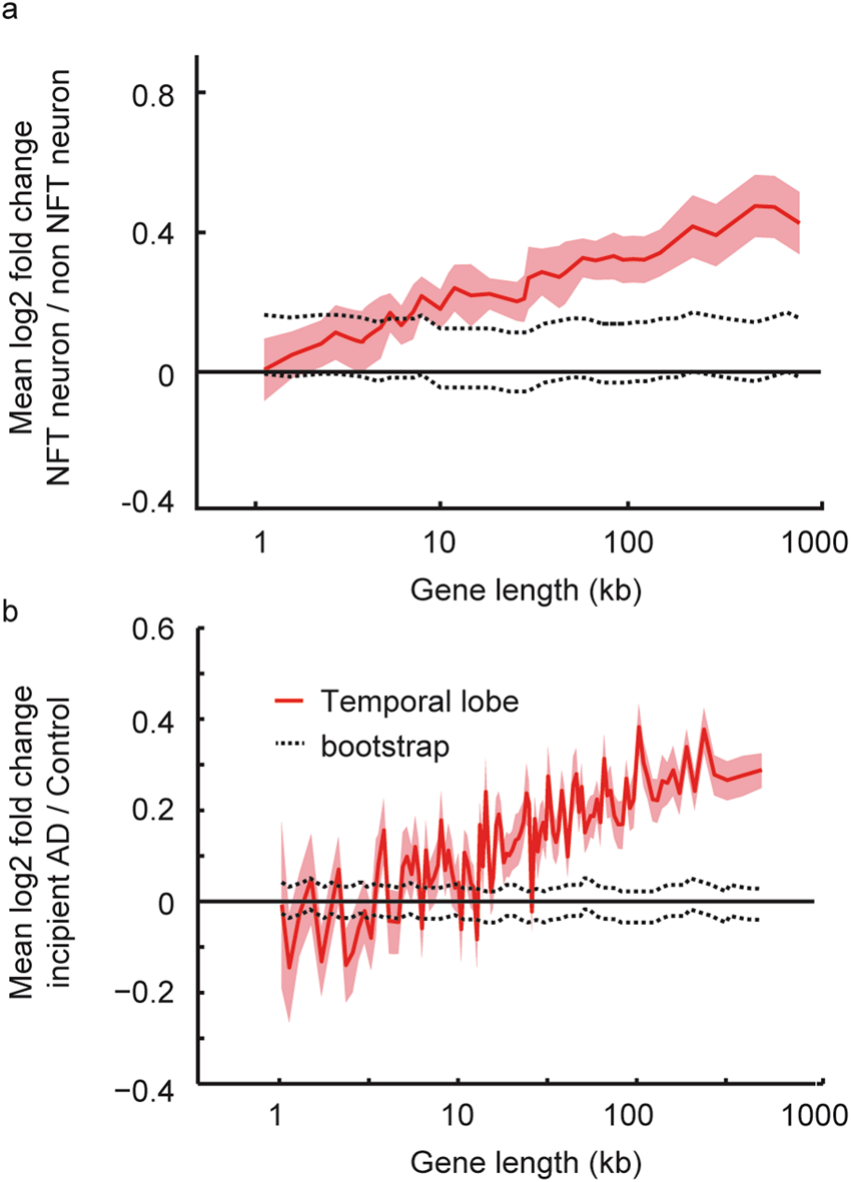
Length-dependent effect on expression in adult human neurons. (**a**) Mean expression changes of genes binned according to gene length for neurons bearing neurofibrillary tangles (NFT) versus non-NFT neurons of the same patient (n = 20 cells from 10 patients). Line indicates mean expression for genes within each 200 gene bin; the ribbons represent the s.e.m. of each bin. Dashed black line represent 95% confidence interval of 100 bootstrap iterations. (**b**) Mean expression of genes binned according to gene length in temporal lobe of patients with pathology but no dementia (n = 8). Line indicates mean expression for genes within each 200 gene bin; the ribbons represent the s.e.m. of each bin. Dashed black line represent 95% confidence interval of 100 bootstrap iterations.

Length-dependent effect on gene expression in AD could be the outcome of loss of neurons, which will lead to a change in ratio between cell populations, or it could be the outcome of a specific change in transcription of long genes in AD neurons, or it could be a mixture between the two. To try to distinguish between these options, we analyzed two additional databases where the neuronal loss component was controlled. Although there is no direct evidence that links neuronal activity and up regulation of long genes, given that molecular functions associated with neuronal activity (such as synaptic formation and neuronal development) are enriched with long genes^6, 15^, we hypothesized that these data sets would show some degree of up regulation of long genes. Hyperactivity of the neurofibrillary tangle afflicted neurons, prior to their degeneration, was shown before^16^. The first data set from ten AD patients (GSE4757^17^) was constructed using laser-captured micro-dissection of neurons that were afflicted with neurofibrillary tangles, and separately neurons that were not afflicted. In this study dead neurons were excluded from the analysis. Neurons with neurofibrillary tangles showed length-dependent increased gene expression compared with neurons without tangles (Fig. 2a). The second database (GSE57152) was from eight controls and eight AD patients that had AD pathology, but were not demented, and showed no neuronal death. We again observed length-dependent increase in gene expression (Fig. 2b), similar to the neuron specific analysis.

Taken together, our results suggest that at early stages of AD, before any apparent major neurodegeneration took place, the main gene length-dependent effect is that of up regulation of long genes. This could also be seen at the cell-specific level in late stages of the disease in non-degenerated neurons. In later disease stages, the effect detected on the whole tissue level is that of down regulation of long genes, since the ratio of the neuronal population compared to non-neuronal population decreases sharply and neurons are the brain cell type that most pronouncedly express long genes.

While previous reports showed this effect in a neurodevelopmental disease, our analysis shows that it also takes place in the context of neurodegeneration. Therefore, analysis of gene length-dependent effects should be considered in future transcriptome studies of neurodegenerative conditions to better map its occurrence and direction. This would also establish reproducibility for observing this effect across a large set of samples, brain regions, disease stages, *etc*. Whether the observed effect is up- or down-stream to the pathological accumulation of protein aggregates (plaques and neurofibrillary tangles) that occurs in AD is an open question, and it is difficult to favor one option over the other based on the results we show here. However, we find it more likely that the gene length-dependent effect is caused by the damaging effects of protein aggregates on neurons, and as such is downstream from protein aggregation. Whether the gene length-dependent effect we observed is protective or damaging to neurons in the context of AD is still to be determined. Further work is also needed to establish the mechanism behind this neuronal misexpression in the AD brain. A reasonable proposed mechanism would be relief of supercoiling during DNA replication and transcription (governed by topoisomerases) since it is implicated in several neurodevelopmental diseases^6^.

## Methods

### Analyzed data

To study RNA expression in relation to gene length, we analyzed the following gene expression datasets available in GEO (https://www.ncbi.nlm.nih.gov/geo/) that have met the following criteria: 1) data were from human brain samples, and 2) profiling methods were unbiased expression profiling of long, polyadenylated genes (which excludes outside microRNA profiling, ChIP-seq, HITS-CLIP, *etc.*). GSE67835, 466 data files, Illumina MiSeq and Illumina NextSeq 500 platforms. GSE36980, 79 data files, Affymetrix Human Gene 1.0 ST Array. GSE56772, 76 data files, Affymetrix HT Mouse Genome 430A Array; this was analyzed by Gabel *et al.* ^1^ where no gene length dependent misexpression was detected. GSE4757, 20 data files, Affymetrix Human Genome U133 Plus 2.0 Array. GSE57152, 16 data files, AB 5500xl Genetic Analyzer. GSE1297, 16 data files, Affymetrix Human Genome U133A Array.

### Data processing steps

The expression data mentioned above were analyzed for expressed genes in each dataset versus the total gene length taken from Ensembl Genome Browser 86 (http://useast.ensembl.org/index.html, updated to September 2016). Only protein coding genes were analyzed, excluding non-coding genes of any kind. After this filtering we were left with between 19,881 and 17,502 genes (depending on the data set) on which we performed the analysis. For microarray data sets, raw CEL files (probe results files) were downloaded from GEO. Probe sets were mapped to genes, with Matlab’s Bioinformatics Toolbox, according to their IDs and GIN library files downloaded from Affymetrix (http://www.affymetrix.com/support/technical/libraryfilesmain.affx). For unambiguous analysis of genes, only transcript cluster IDs that map to single RefSeq genes were included. Next, expression values for genes with multiple transcript clusters were taken as the average log2 expression value across all transcript clusters of a gene. For RNA-seq datasets, files of number of mapped reads per gene were downloaded from GEO (for example GSE67835_RAW.tar for study GSE67835), these were normalized by the total length of the gene’s exons and so transformed to averaged exon density values. For each data set, the samples were quantile normalized between them, to allow for confident comparison between samples (quantile normalization leads to sample distributions with similar statistical characteristics). At this point expression values were averaged per gene of each condition separately following by calculation of the log fold change between conditions, with variance taken as the sum of variances for each condition separately. Next, genes were sorted by their lengths and then binned to groups of 200 consecutive genes with a 40 gene step between the groups. Averages for the measurements described above (log of fold change) were calculated and presented across these gene groups as a function of their averaged length.

### Bootstrap analysis

Because no statistical framework yet exists for calling significance for a gene length dependent effect, we used bootstrap analysis; random resampling with replacement to build the null hypothesis to which the actual data were compared. We shuffled across expression values of genes (estimated as described above) and their lengths, so that in each iteration there would be an ‘unnatural’, random pairing between an expression value and a gene length value. For each bootstrap iteration we performed the 200 gene group binning, averaged across the groups (as described above) and collected the line describing the expression to length relationship. This procedure was done 100 times and the 95% confidence interval across these 100 iterations is presented in the figures overlaid with the real results.
